# Current prospects of hereditary adrenal tumors: towards better clinical management

**DOI:** 10.1186/s13053-024-00276-6

**Published:** 2024-03-26

**Authors:** Akihiro Ohmoto, Naomi Hayashi, Shunji Takahashi, Arisa Ueki

**Affiliations:** 1 Division of Medical Oncology, Cancer Institute Hospital of Japanese Foundation for Cancer Research, Tokyo, 1358550 Japan; 2 Human Oncology and Pathogenesis Program, Memorial Sloan Kettering Cancer Center, 417 East 68th Street, New York, NY 10065 USA; 3 Division of Genomic Medicine, Cancer Institute Hospital of Japanese Foundation for Cancer Research, Tokyo, 1358550 Japan; 4 Division of Clinical Genetic Oncology, Cancer Institute Hospital of Japanese Foundation for Cancer Research, Tokyo, 1358550 Japan

**Keywords:** Pheochromocytoma, Paraganglioma, Hereditary tumors, Phenotype, Universal multi-gene germline panel, Personalized medicine

## Abstract

Adrenocortical carcinoma (ACC) and pheochromocytoma/paraganglioma (PPGL) are two rare types of adrenal gland malignancies. Regarding hereditary tumors, some patients with ACC are associated with with Li-Fraumeni syndrome (LFS), and those with PPGL with multiple endocrine neoplasia type 2. Recent studies have expanded this spectrum to include other types of hereditary tumors, such as Lynch syndrome or familial adenomatous polyposis. Individuals harboring germline *TP53* pathogenic variants that cause LFS have heterogeneous phenotypes depending on the respective variant type. As an example, R337H variant found in Brazilian is known as low penetrant. While 50–80% of pediatric ACC patients harbored a LFS, such a strong causal relationship is not observed in adult patients, which suggests different pathophysiologies between the two populations. As for PPGL, because multiple driver genes, such as succinate dehydrogenase (SDH)-related genes, *RET*, *NF1*, and *VHL* have been identified, universal multi-gene germline panel testing is warranted as a comprehensive and cost-effective approach. PPGL pathogenesis is divided into three molecular pathways (pseudohypoxia, Wnt signaling, and kinase signaling), and this classification is expected to result in personalized medicine based on genomic profiles. It remains unknown whether clinical characteristics differ between cases derived from genetic predisposition syndromes and sporadic cases, or whether the surveillance strategy should be changed depending on the genetic background or whether it should be uniform. Close cooperation among medical genomics experts, endocrinologists, oncologists, and early investigators is indispensable for improving the clinical management for multifaceted ACC and PPGL.

## Introduction

Adrenocortical carcinoma (ACC) and pheochromocytoma are the two types of adrenal gland malignancies. Extra-adrenal paraganglioma is pathologically categorized into the same entity as pheochromocytoma, and these two diseases are collectively termed pheochromocytoma/paraganglioma (PPGL) [1]. ACC and PPGL are the major adrenal malignancies; however, they rarely occur. According to the Rare Cancers in Europe (RARECARE) project data, the annual incidences of ACC, malignant pheochromocytoma, and paraganglioma are 0.22, 0.04, and 0.02 per 100,000 individuals per year, meeting the criteria for rare malignancies (< 6 per 100,000) [2]. While surgical resection is generally recommended for localized ACC/PPGL, the approved systemic chemotherapy for advanced cases remains the conventional cytotoxic regimen (mitotane alone or a combination of etoposide, doxorubicin, and cisplatin plus mitotane for ACC; a combination of cyclophosphamide, vincristine, and dacarbazine [CVD]; or temozolomide alone for PPGL) [1]. Therefore, novel therapeutic strategies are required.

It is well known that some individuals with specific genetic pathogenic/likely pathogenic variants develop ACC or PPGL. According to a large genomic analysis of pediatric cancers, the percentage of pathogenic germline variant carriers among all types of malignancies was the highest in ACC (approximately 50%) [3]. Representative hereditary genetic syndromes relevant to ACC and PPGL include Li-Fraumeni syndrome (LFS) and multiple endocrine neoplasia type 2 (MEN2), respectively [4]. Recent investigations have broadened this spectrum to include a wider range of syndromes, such as Lynch syndrome. With regard to therapeutic interventions after disease onset, clinical development through elucidating molecular mechanisms is promising. Another focus is on early diagnosis through routine surveillance. There is no established treatment approach yet for hereditary adrenal tumors, unlike well-known hereditary syndromes such as breast and ovarian cancer or Lynch syndrome.

To aid clinical decision, this manuscript provides an overview of the latest information about genetic predisposition syndromes associated with ACC and PPGL, the molecular pathways that could result in novel treatments, and discusses prospects for treatment and surveillance. In this review, database searches were conducted using PubMed/MEDLINE. The following keywords were used for literature retrieval: ((adrenal tumor) OR (adrenal carcinoma) OR (pheochromocytoma) OR (paraganglioma)) AND (hereditary cancer). Regarding novel agents for adrenal tumors, we added manual searches.

## Hereditary malignancies associated with adrenal tumors

The National Comprehensive Cancer Network (NCCN) guidelines for neuroendocrine and adrenal tumors include LFS, Lynch syndrome (LS), multiple endocrine neoplasia type 1 (MEN1), and familial adenomatous polyposis (APC) as hereditary cancer predisposition syndromes associated with ACC [4]. Hereditary PPGL syndrome and MEN type 2 (MEN2) were also mentioned [4]. The clinical and genomic features of representative hereditary tumors associated with adrenal tumors are presented in Table 1**,** and the available surveillance strategies proposed in the guidelines or expert statements are listed in Table 2.

**Table 1:** Clinical and genomic features in hereditary tumors linked with adrenal tumor

Hereditary tumor	Relevant adrenal tumor	Causative gene	Mode of inheritance	Incidence of respective syndrome	Median onset of adrenal tumor	Percentage of patients who were diagnosed with adrenal tumor among hereditary syndrome individuals	Genotype/phenotype relationship	References
LFS	ACC	*TP53*	Autosomal dominant	1: 5,000-20,000	3 years	13% (27% in children cohort; 3% in adult cohort)	Yes	[5, 6]
LS	ACC	MMR genes (*MLH1*, *MSH2*, *MSH6*, and *PMS2*)	Autosomal dominant	1: 660-2,000	64 years	0.5%	Unknown	[7]
FAP	ACC	*APC*	Autosomal dominant	1: 8,000-18,000	NA	NA	Unknown	[8]
BWS	ACC	Defects on chromosome 11p15.5	Not applicable	1: 13,700	10 months	0.3%	Unknown	[9]
MEN2	PPGL	*RET*	Autosomal dominant	1: 35,000	39 years	37%	Yes	[10]
VHL	PPGL	*VHL*	Autosomal dominant	1: 36,000–91,000	27 years (mean)	19%	Yes	[11, 12]
NF1	PPGL	*NF1*	Autosomal dominant	1: 2,500-3,000	41 years; 42 years	3%	Unknown	[13, 14]

*LFS* Li-Fraumeni syndrome: *LS* Lynch syndrome: *FAP* familial adenomatous polyposis: *BWS* Beckwith-Wiedemann syndrome: *MEN2* multiple endocrine neoplasia type 2: *VHL* von Hippel-Lindau disease

*NF1* Neurofibromatosis type 1: *ACC* adrenocortical carcinoma: *PPGL* pheochromocytoma/paraganglioma: *NA* not available

**Table 2:** Surveillance strategies proposed by guidelines or expert statements

Hereditary tumor	Surveillance method of adrenal tumor	Guidelines/ statements	References
LFS	For children: abdominal and pelvic ultrasound, and alternative blood test (total testosterone, dehydroepiandrosterone, and androstenedione) (every 3–4 months)	Japanese LFS medical guidelines; Toronto protocol	[15, 16 ]
LS	NA	NA	
FAP	NA	NA	
MEN2	Annual measurement of plasma free or 24-hour urine fractionated metanephrine/normetanephrine	NCCN guidelines	[4]
VHL	Plasma-metanephrine/normetanephrine (every year from age 5); abdominal MRI (every 2-years from age 15, no upper limit of age)	VHL Alliance; Danish guidelines	[17, 18]
NF1	Biochemical testing in individual who has raised blood pressure	ERN GENTURIS guidelines	[19]
Hereditary PPGL syndrome	Blood pressure monitoring at all medical visits, annual measurement of plasma free metanephrine or 24-hour urine for fractionated metanephrine, and whole body MRI every 2-3 years (from age 6–10 for patients with *SDHB* mutations and age 10–15 for patients with all other *SDHx* mutations)	NCCN guidelines	[4]

*LFS* Li-Fraumeni syndrome: *LS* Lynch syndrome: *FAP* familial adenomatous polyposis: *MEN2* multiple endocrine neoplasia type 2: *VHL* von Hippel-Lindau disease: *NF1* Neurofibromatosis type 1: *PPGL* pheochromocytoma/paraganglioma: *NA* not available: *NCCN* National Comprehensive Cancer Network: *ERN GENTURIS* European Reference Network on Genetic Tumor Risk Syndromes

### ACC

#### Li-Fraumeni syndrome

LFS is a genetically predisposed syndrome involving *TP53* germline pathogenic variants (g*TP53*) and is inherited in an autosomal-dominant manner. *TP53* is a tumor suppressor gene that plays a central role in mediating the cellular response to genotoxic stress and oncogene activation and activates pathways involved in cell cycle arrest and DNA damage repair [20]. The incidence of LFS is as frequent as one in 5000–20,000 individuals [21]. According to the National Cancer Institute’s referral-based longitudinal LFS study, individuals with LFS have a nearly 24-fold higher incidence of cancer than the general population [22]. ACC is included in the core tumors of LFS, along with bone and soft tissue sarcomas and breast and brain cancers [15]. The Chompret Criteria designates ACC along with choroid plexus tumor or rhabdomyosarcoma of the embryonal anaplastic subtype [15]. The tumor distribution in affected g*TP53* carriers differs between children and adults. According to a French database analysis, ACC was observed in 13% of affected g*TP53* carriers (27% in children, *n* = 132; 3% in adults, *n* = 219; total *n* = 322) [5]. A US study including 286 individuals harboring g*TP53* showed that the cumulative incidence of any cancer was 50% by age 31 among females and by age 46 among males; it approached 100% by age 70 in both groups [23]. Among the 403 cancer diagnoses, the most common malignancy type was breast cancer, and five patients with ACC were diagnosed at age 17 or earlier [23]. A study of 88 children with ACC detected g*TP53* in 50% of the cases, regardless of family history [24]. Similar to the French study above, mutations were prevalent in young patients (51% at 0–4 years, 73% at 4–12 years, 29% at 12–20 years, and 6% at 20–30 years). A single-center prospective analysis in the US showed that 7.5% of 53 unselected patients with ACC had g*TP53* [25]. In the clinical sequencing data from the Memorial Sloan Kettering Cancer Center, four of 1566 (0.2%) individuals harbored g*TP53* [26]. According to an analysis by the Japanese LFS Special Committee, 68 individuals from 48 families harboring g*TP53* were found [6]. Nine of these 68 patients (13%) developed ACC at a median of 3.0 years (range, 0–31 years). ACC onset was the earliest among all tumor types (median = 26.0 years). Furthermore, there are endemic geographic areas for pediatric ACC. In southern Brazil, the incidence of ACC is 10–15 times higher than the global incidence, and most patients have the g*TP53* variant R337H [27]. In a large population-based screening study including 171,649 newborns from Paraná State (Brazil), the R337H variant was identified in 461 individuals (0.3%) [28].

One focus is on the genotype-phenotype relationship in LFS. In a study by Wasserman et al., g*TP53* was found in a wide range of positions beyond hotspots. Patients harboring alleles with severe loss-of-function (LOF) mutations (e.g., Y163C, H193P, C275X, and E285V) have a strong family history, whereas those harboring alleles without LOF (e.g., R337H) have a relatively weak family history [24]. Bougeard et al. revealed that the age at first tumor onset varied depending on the type of g*TP53* alteration [5]. Briefly, dominant-negative missense mutations drastically alter the transcriptional response to DNA damage that occurred during childhood. In contrast, the onset of LOF mutations and genomic rearrangements are mostly observed in adults. Additionally, heterogeneity in onset was observed among variants, where individuals harboring the R337H variant mostly developed the first tumor in around 40 years or later. The aforementioned US observational cohort study showed that g*TP53* LOF variants, regardless of their dominant-negative effect (DNE), were associated with an earlier onset of first and second cancers than non-LOF variants, regardless of DNE [22]. According to another pooled analysis of 427 g*TP53* carriers who underwent multi-gene panel testing and 154 g*TP53* carriers with *TP53* single-gene testing, carriers of truncated and hotspot variants tended to present with LFS cancers and had a shorter time to first cancer diagnosis than carriers of missense variants [29]. These data highlight the functional significance of these variants. On the other hand, it should be noted that the evidence about genotype-phenotype relationship is not robust to assist with clinical management (risk stratification or personalized screening) of *TP53* carriers.

Regarding the surveillance of children with ACC, the Toronto Protocol implemented in Canada and the USA and the Japanese LFS medical guidelines recommend abdominal and pelvic ultrasound every 3–4 months and alternative blood tests (total testosterone, dehydroepiandrosterone, and androstenedione) every 3–4 months [15, 16]. However, no definite recommendations exist for adult ACC, partly because the causality is not always strong in adults. For LFS, priority should be placed on surgical or ablative treatment, and radiotherapy should be avoided when possible; non-genotoxic chemotherapies have also been adopted [30]. Moreover, whole-body MRI is recommended as a regular screening method [30, 31].

#### Lynch syndrome

LS derived from pathogenic germline variants (PGV) of DNA mismatch repair (MMR) genes (*MLH1*, *MSH2*, *MSH6*, and *PMS2*) or *EPCAM* deletion leading to the silencing of *MSH2* are inherited in an autosomal-dominant manner [32]. LS causes several types of malignancies, such as colorectal and endometrial cancers, and clinical information is gradually accumulating in the context of ACC [32]. A Spanish LS cohort analysis included 634 individuals from 220 families with LS and identified three (0.5%) patients with ACC [7]. These patients harbored *MSH2* PGV and presented with MSH2 and MSH6 protein loss. Raymond et al. analyzed 84 patients with ACC who underwent genetic counseling and identified three patients (3%) with family histories suggestive of LS [33]. All three families had MMR PGVs (*MSH2* p.R711X, *MLH1* p.L749P, and *MSH6* p.S714C). The prevalence of LS among ACC cases (3%) exhibited in this study was comparable to colorectal cancer or endometrial cancer [34]. Furthermore, there have been case reports of ACC harboring MMR PGVs [35–39]. However, the clinical evidence for routine ACC screening is insufficient for individuals with LS.

#### Familial adenomatous polyposis (FAP) and Beckwith-Wiedemann syndrome (BWS)

FAP and BWS are hereditary syndromes associated with adult and childhood ACC, respectively [40]. In both syndromes, Wnt/β-catenin pathway regulation contributes to the oncogenesis of adrenocortical tumors (ACT) [8]. In contrast to the LFS, information on the link between FAP/BWS and ACC is limited. Gaujoux et al. identified four ACT, including one ACC, in three individuals with FAP and showed that ACT tumorigenesis was mediated by biallelic APC inactivation [8]. BWS is a genetic disorder characterized by overgrowth. This syndrome is caused by defects in chromosome 11p15.5, which result in the overexpression of insulin-like growth factor 2 (IGF2) [41]. Several molecular mechanisms including imprinting control region 1 (IC1) gain of methylation or paternal uniparental disomy of 11p15 induce overexpression of IGF2 [42]. Any malignancy occurred in 5% of patients with BWS, dependent on the molecular mechanisms [43]. Cöktü et al. analyzed 321 individuals with BWS and identified 13 cases of cancer (33-fold increased risk compared with expected number of cases) [9]. The major cancer types were hepatoblastoma (*n* = 6) and nephroblastoma (Wilms tumor) (*n* = 4), and ACC (*n* = 1). An international consensus statement for the clinical management of BWS mentioned a screening strategy using clinical evaluation, adrenal ultrasound, and evaluating serum dehydroepiandrosterone sulfate levels every 4–6 months [44]. However, as presented here, the incidence of ACC is low in patients with BWS, and there are no data on the utility of such screening strategies.

### PPGL

#### Multiple endocrine neoplasia type 2 (MEN2)

MEN2 is an autosomal dominant genetic syndrome with germline *RET*-activating alterations and can be further classified into MEN2A and MEN2B. MEN2 accounts for approximately 5% of PPGL cases [45], with an incidence of one in 35,000 individuals. Although the penetrance of medullary thyroid carcinoma (MTC) is approximately 100%, PPGL occurs in up to 50% of individuals [46], the genotype-phenotype correlation of MEN2 is noteworthy. The penetrance and onset age of MEN2 depend on the type of *RET* variants present [47]. The penetrance of PPGL is particularly high in the *RET* p.D631Y, p.C634F/G/R/S/W/Y, p.A883F, and M918T variants. Those with the M918T variant have the highest risk of aggressive MTC [48]. According to an analysis by the MEN Consortium of Japan, 144 (37%) out of 390 individuals with MEN2 developed PPGL [10]. In that study, the penetrance of pheochromocytoma was high in *RET* pathogenic variants in codons 918 (100% by age 56) and 634 (88% by age 77); the rate was lower in other variants (< 32% for in codons 611, 618, 620, and 768). Consistent with this, Qi et al. reported that PPGL penetrance was higher in individuals harboring *RET* p.C634F/G/R/S/W/Y variants in exon 11 than in those harboring pathogenic variants in exon 10 (e.g., p.C609R, p.C611F/Y, p.C618G/R/S/Y, and p.C620R/S) [49]. Thosani et al. analyzed 319 individuals with MEN2 at the MD Anderson Cancer Center and compared the clinical features between 59 PPGL patients with *RET* codon 634 variants and 48 individuals without PPGL but harbored codon 634 variants [50]. In their study, the clinical stage distribution of MTC at the initial diagnosis and overall survival were not significantly different between the two groups. NCCN guidelines recommend surveillance for PPGL using plasma-free or 24-hour urine-fractionated metanephrine and normetanephrine levels [4]. However, these guidelines do not include imaging modalities, such as computed tomography or ultrasound.

### von Hippel-Lindau disease (VHL)

VHL is an autosomal dominant genetic syndrome characterized by hemangioblastoma of the retina, central nervous system and renal cell carcinomas, neuroendocrine pancreatic tumors, endolymphatic sac tumors, and pheochromocytoma [18]. *VHL* is a tumor suppressor gene that causes this syndrome. The incidence of VHL is one in 36,000–91,000 individuals. Regarding PPGL, the estimated lifetime risk is 10–25% [11]. According to a systematic review and meta-analysis of VHL-associated PPGL that included 4263 VHL cases, the pooled frequency of PPGL was 19.4% [12]. VHL is classified into types 1 and 2; only patients with type 2 VHL develop PPGL. A genotype-phenotype relationship was observed between type 1 and type 2 VHL. While protein-truncating mutations are detected in type 1 VHL, missense mutations that generally do not affect protein structure are unique to type 2 VHL [11, 51].

Regarding tumor surveillance for VHL-associated PPGL, the VHL Alliance recommends annual plasma-free metanephrine or urinary-fractionated metanephrine monitoring from 5 years of age, abdominal MRI every 2 years from the age of 15 years, and annual blood pressure and pulse monitoring from 2 years of age [17]. Consistently, the Danish guidelines propose the annual measurement of plasma metanephrine/normetanephrine from age 5 and imaging of the abdomen every 2 years from age 15 [18].

#### Neurofibromatosis type 1 (NF1)

NF1 is a dominantly inherited genetic disorder caused by PGV in the tumor suppressor gene *NF1*. Individuals with NF1 characteristically develop benign and malignant tumors of the central and peripheral nervous systems (gliomas of the optic pathway, glioblastomas, and malignant peripheral nerve sheath tumors) [52]. This syndrome is also associated with an increased incidence of other malignancies (gastrointestinal stromal tumors, breast cancer, leukemia, duodenal carcinoid tumors, rhabdomyosarcomas, and pheochromocytoma) [52]. The prevalence of NF1 is one in 2500–3000 births, and 5–7% of individuals with NF1 are estimated to develop PPGL in their lifetime. Several studies have described the clinical features of NF-1-associated PPGL. Gruber et al. reported that the prevalence of PPGL in patients with NF1 was 2.9% (41/1415) [13]. The median age at diagnosis was 41 years, and metastatic or recurrent disease was detected in 7% of cases. According to a US single-center retrospective analysis of 17 patients with NF-1-associated PPGL, the average age at diagnosis was 42 years, and two patients had metastatic disease [14]. Al-Sharefi et al. reviewed 27 patients with NF1 who developed PPGL and reported that PPGL diagnosis was incidental in 48% of the cases [53]. The European Reference Network on Genetic Tumor Risk Syndromes (ERN GENTURIS) tumor surveillance guidelines state that biochemical testing for PPGL should be conducted in any individual with NF1 who has elevated blood pressure, while routine biochemical screening for PPGL is not recommended except in pregnant women [19].

Finally, according to the American College of Medical Genetics and Genomics (ACMG) statement, hereditary PPGL syndrome characterized by succinate dehydrogenase (SDH)-related gene mutations is categorized as an independent type of genetic predisposition syndrome [54]. Their molecular features are discussed in the following sections. Regarding surveillance, annual measurements of plasma-free metanephrine or 24-hour urine-fractionated metanephrine and whole-body MRI every 2–3 years are recommended. Notably, the surveillance start age differs between *SDHB*-mutated individuals (6–10 years) and other *SDHx*-mutated individuals (10–15 years) [4].

## Molecular features and druggable mutated genes for adrenal tumors

### ACC

**Fig. 1:**
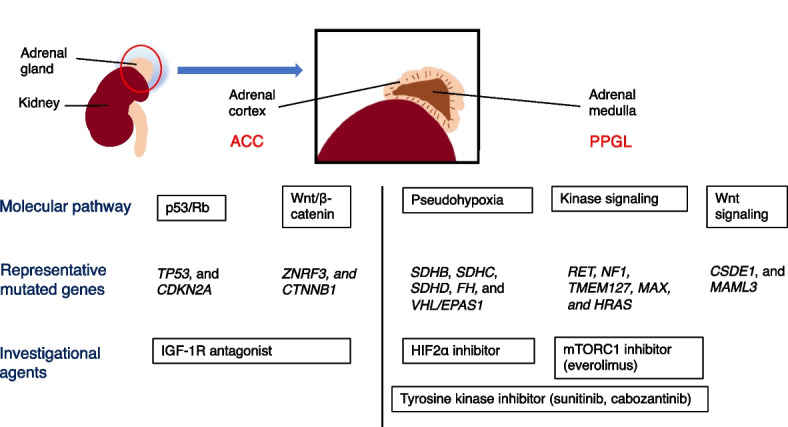
Molecular features and molecularly targeted agents for druggable genes in adrenal tumors. ACC from the adrenal cortex and PPGL from the adrenal medulla have different molecular features. The major molecular cascades are p53/Rb and Wnt/β-catenin pathways for ACC, and pseudohypoxia, kinase signaling, and Wnt signaling pathways for PPGL. The treatment types differ depending on the mutated genes present, such as HIF-2α inhibitors for pseudohypoxia-associated PPGL

A comprehensive genomic analysis of 91 ACC cases revealed its genomic landscape [55]. The two major signaling cascades involved in ACC oncogenesis are the p53/Rb and Wnt/β-catenin pathways (Fig. 1). Deactivating mutations in *TP53*, *CDKN2A*, and *ZNRF3* and activating mutations in *CTNNB1* were detected in 21, 16, 19, and 16% of cases, respectively. *CDKN2A* participates in the p53/Rb pathway, while *ZNRF3* and *CTNNB1* participate in the β-catenin pathway. In addition, alterations in histone modification genes (*MLL*, *MLL2*, and *MLL4*) and chromatin remodeling genes (*ATRX* and *DAXX*) were detected in 22% of cases. Another study, which included 45 ACC cases, identified *ZNRF3* alterations in 21% [56].

Although the genomic features of ACC have been gradually uncovered, there are currently no approved molecular-targeted agents for ACC. The NCCN and European Society of Medical Oncology (ESMO) guidelines only recommend conventional cytotoxic regimens for advanced ACC [1, 4]. Under the current status, transcriptional analysis showed that IGF2 expression is enhanced in 80–90% of ACC cases. This finding suggests the utility of type 1 IGF receptor (IGF-1R) antagonists for ACC, in which IGF-2 is the ligand of IGF-1R [57]. However, a phase 3 trial of linsitinib, a dual inhibitor of IGF-1R and the insulin receptor, did not demonstrate a survival benefit over the placebo for patients with locally advanced or metastatic ACC [58].

Unfortunately, advances in genomic medicine have not always improved the outcomes of ACC. Current issues include inadequate patient selection and pharmacological interactions between novel agents and already-approved mitotanes [59]. Considering the heterogeneous molecular features of ACC, personalized treatment based on individual genomic information is essential. Immune checkpoint inhibitors are promising options for the treatment of LS-associated ACC. According to a phase 2 trial of pembrolizumab in 39 patients with advanced ACC, the overall response rate (ORR) and disease control rate were 23 and 52%, respectively [60]. Unexpectedly, genetic alterations, tumor mutation burden, tumor programmed death-ligand 1 expression, and microsatellite-high and/or mismatch repair-deficient status were not associated with ORR. In addition, Wnt/β-catenin signaling is a major molecular pathway in ACC. Hence, clinical investigations using Wnt signaling inhibitors, such as those ongoing for colorectal cancer, are warranted for ACC in the future [61].

### PPGL

Owing to recent advances in molecular classification methods, universal germline panel testing in patients with PPGL has been actively discussed. This information is helpful not only for the active surveillance of mutation carriers but also for treatment selection. PPGL pathogenesis is broadly divided into the pseudohypoxia pathway, kinase signaling, and Wnt signaling (Fig. 1) [62]. The following mutated genes were included in each pathway: (**pseudohypoxia signaling**) SDH-related genes such as *SDHB*, *SDHD*, and *SDHC* encoding succinate dehydrogenase subunits, *FH,* and *VHL/EPAS1*; (**kinase signaling**) *RET*, *NF1*, *TMEM127*, *MAX*, and *HRAS*; and (**Wnt signaling**) *CSDE1* and *MAML3*. Regarding mutation distribution, 40% of PPGL cases harbor PGV and 30% harbor somatic mutations in predisposition genes [63]. Amar et al. sequenced five major PPGL susceptibility genes (*RET*, *VHL*, *SDHB*, *SDHD*, and *SDHC*) in blood samples from 314 patients with PPGL [45] and identified heterozygous PGV in 73 patients (23%) and diagnosed NF1 in 13 patients (4%). In total, 86 patients (27%) were diagnosed with hereditary PPGL. The prevalence of PGVs was 5% for *RET*, 8% for *VHL*, 7% for *SDHB*, and 4% for *SDHD*. Remarkably, 12% of seemingly sporadic cases without a family history and syndromic presentation harbored PPGL-associated PGVs. Patients with germline *SDHB* pathogenic variants had larger, more frequent extra-adrenal and malignant PPGL. Yonamine et al. also showed that 32% of 370 patients with PPGL had PGVs in seven susceptibility genes (*MAX*, *SDHB*, *SDHC*, *SDHD*, *TMEM127*, *VHL*, and *RET*), with a prevalence of 25% in seemingly sporadic cases [64]. The incidence of metastatic PPGL was high in *SDHB* carriers (37%). According to a large cohort study assessing 1727 individuals suspected to have hereditary PPGL and underwent multi-gene panel testing, 28% had PGVs, of which the common causative genes were *SDHB* (40%), *SDHD* (21%), *SDHA* (10%), *VHL* (8%), *SDHC* (7%), *RET* (4%), and *MAX* (4%) [65]. In a prospective cohort study based on the European-American-Asian PPGL Registry, 6% individuals (58/972) had PGVs, including 29 with *SDHA* PGVs, 20 in *TMEM127*, and eight in *MAX,* and one in *SDHAF* [66].

Regarding genetic heterogeneity in PPGL, targeted next-generation sequencing (NGS) custom panels using blood samples are considered the gold standard for genetic diagnosis [63]. The PPGL Study Group stated that targeted NGS is a favored approach in terms of turnaround time, autonomy of individual laboratories, assay flexibility, scalability, bioinformatics needs, data storage, and interpretation, regardless of the required cost [67]. Based on current evidence, they advocated three NGS options: a basic panel of 10 genes (*FH*, *MAX*, *NF1*, *RET*, *SDHA*, *SDHB*, *SDHC*, *SDHD*, *TMEM127*, and *VHL*), an extended panel of 15 genes (the 10 genes in the basic panel plus *EGLN1*/*PHD2*, *EPAS1*, *KIF1B*, *MET*, and *SDHAF2*), and a comprehensive panel of 27 genes (the 15 genes in the extended panel plus *ATRX*, *BRAF*, *CDKN2A*, *EGLN2/PHD1*, *FGFR1*, *H3F3A*, *HRAS*, *IDH2*, *KMT2D*, *MDH2*, *MERTK*, and *TP53*). While the basic and extended panels aim to detect PGVs in blood samples, a comprehensive panel incorporates somatic mutation detection in tumor samples.

Because precision oncology with NGS is pervasive in clinical practice, issues regarding incidental findings are inevitable. Several mutations detected in tumor samples suggest the possibility of genetic predisposition syndromes. The ACMG statements published in 2013 and 2017 recommend the return of incidental findings in clinical sequencing, in which four genes (*SDHD*, *SDHAF2*, *SDHC*, and *SDHB*) are specified for hereditary PPGL syndrome [68, 69]. In the latest version (version 3.1), published in 2022, the number of target genes for hereditary PPGL syndrome was increased to six (*SDHD*, *SDHAF2*, *SDHC*, *SDHB*, *MAX*, *TMEM127*) [54]. Identifying these potentially actionable genes is essential for active surveillance and therapeutic interventions.

**Fig. 2:**
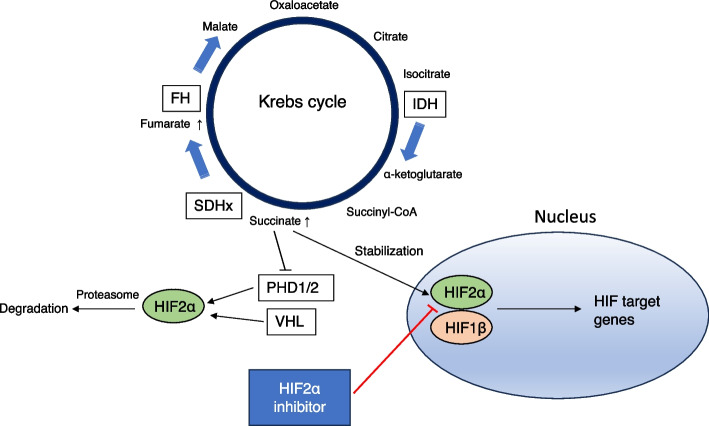
Pseudohypoxia-related pheochromocytoma/paraganglioma (PPGL) and action mechanisms of hypoxia-inducible factor 2-alpha (HIF-2α) inhibitors. The HIF pathway is activated through the following processes: i) loss-of-function mutations in genes encoding molecules in the Krebs cycle (e.g., *SDHx* or *FH*), leading to succinate accumulation; ii) loss-of-function mutations in *PHD1/2* or *VHL*, suppressing HIF-2α degradation. After succinate accumulation, HIF-2α/HIF-1β heterodimer formation following HIF-2α stabilization occurs in the nucleus, which activates the transcription of HIF target genes related to oncogenesis (e.g., angiogenesis- or cell cycle-related genes). HIF-2α inhibitors decrease the dimerization of HIF-2α with HIF-1β and suppress HIF target gene activation

In the context of novel treatments, pseudohypoxia-related PPGL is a promising candidate for blocking vascular endothelial growth factor or hypoxia-inducible factor 2-alpha (HIF-2α) signaling (Fig. 1). The first placebo-controlled phase 2 trial of the tyrosine kinase inhibitor (TKI) sunitinib (FIRSTMAPPP trial) demonstrated a higher progression-free survival (PFS) rate at 12 months in the sunitinib group (36% vs. 19%) [70]. Interestingly, the ORR of 12 patients with *SDHB* mutations was promising (50%). HIF-2α is activated by sensitizing mutations in SDH-related genes (*SDHA*, *SDHB*, *SDHC*, *SDHD*, and *SDHAF2*), *VHL*, and *EPAS1*, as a HIF-2α-encoding gene [71]. The selective HIF-2α inhibitor belzutifan has been approved in the US to treat VHL-driven cancers. A phase 2 trial of belzutifan in patients with renal cell carcinoma associated with VHL exhibited an ORR of 49% [72]. A phase 2 study of belzutifan for PPGL, pancreatic neuroendocrine tumors, and VHL-associated tumors is currently underway (NCT04924075). In addition, mTOR complex 1 inhibitors for kinase signaling PPGL, such as everolimus, or other TKIs, such as cabozantinib, are promising agents [73]. Selective RET TKIs are also promising for treating patients with *RET* mutations. Regarding PPGL, Mweempwa et al. reported a patient with *RET*-*SEPTIN9* fusion who responded to the selective RET inhibitor selpercatinib upon treatment [74]. Pseudohypoxia-related PPGL and the mechanisms of action of the corresponding HIF-2α inhibitors are shown in Fig. 2 [73, 75].

### Conclusions and future prospects

In this manuscript, we reviewed representative genetic predisposition syndromes associated with adrenal tumors (ACC and PPGL) and discussed their surveillance and therapeutic interventions. In particular, the genotype-phenotype relationship and clinical development of molecular-targeted agents based on genomic information were highlighted. A genotype-phenotype relationship indicated that ACC was associated with LFS, whereas PPGL was associated with MEN2 or VHL. Overall, the clinical behavior and biological features of ACC and PPGL are different, although both diseases develop from the adrenal gland. Our retrospective single-institution analysis suggested a significant difference in overall survival (OS) between patients with ACC who received mitotane-based regimens and those with PPGL who received the CVD regimen (median OS, 7.2 months vs. 4.4 years) [76, 77].

Several topics should be addressed in future research. First, it remains unknown whether clinical characteristics, including prognosis, differ between cases derived from genetic predisposition syndromes and sporadic cases. Second, whether the surveillance strategy should be changed depending on the genetic background or whether it should be uniform has not been determined. As shown in Table 1, the age at onset of adrenal tumors is heterogeneous among genetic syndromes; hence, the surveillance strategy will be affected. In the context of novel PPGL treatments, the PFS benefit of sunitinib is valuable in advancing the clinical development of this rare malignancy. Treatment stratification based on molecular profiles, such as HIF-2α inhibitors, is also expected. However, available molecular information does not affect the clinical development of ACC. Close cooperation among medical genomics experts, endocrinologists, oncologists, and early investigators is indispensable for improving the clinical management of patients with these multifaceted diseases.

## Data Availability

Not applicable.

## Abbreviations

ACC: adrenocortical carcinoma
PPGL: pheochromocytoma/paraganglioma
IGF2: insulin-like growth factor 2
HIF2α: hypoxia-inducible factor 2-alpha
mTORC1: mammalian target of rapamycin complex1
